# Enhancing PLP-Binding Capacity of Class-III ω-Transaminase by Single Residue Substitution

**DOI:** 10.3389/fbioe.2019.00282

**Published:** 2019-10-18

**Authors:** David Roura Padrosa, Raphael Alaux, Phillip Smith, Ingrid Dreveny, Fernando López-Gallego, Francesca Paradisi

**Affiliations:** ^1^School of Chemistry, University of Nottingham, Nottingham, United Kingdom; ^2^Centre for Biomolecular Sciences, University of Nottingham, Nottingham, United Kingdom; ^3^Instituto de Síntesis Química y Catálisis Homogénea, Zaragoza, Spain; ^4^ARAID Foundation, Zaragoza, Spain; ^5^Department of Chemistry and Biochemistry, University of Bern, Bern, Switzerland

**Keywords:** pyridoxal phosphate, protein stability, protein engineering, biocatalysis, transaminase

## Abstract

Transaminases are pyridoxal-5′-phosphate (PLP) binding enzymes, broadly studied for their potential industrial application. Their affinity for PLP has been related to their performance and operational stability and while significant differences in PLP requirements have been reported, the environment of the PLP-binding pocket is highly conserved. In this study, thorough analysis of the residue interaction network of three homologous transaminases *Halomonas elongata* (HeTA), *Chromobacterium violaceum* (CvTA), and *Pseudomonas fluorescens* (PfTA) revealed a single residue difference in their PLP binding pocket: an asparagine at position 120 in HeTA. N120 is suitably positioned to interact with an aspartic acid known to protonate the PLP pyridinium nitrogen, while the equivalent position is occupied by a valine in the other two enzymes. Three different mutants were constructed (HeTA-N120V, CvTA-V124N, and PfTA-V129N) and functionally analyzed. Notably, in HeTA and CvTA, the asparagine variants, consistently exhibited a higher thermal stability and a significant decrease in the dissociation constant (*K*_*d*_*)* for PLP, confirming the important role of N120 in PLP binding. Moreover, the reaction intermediate pyridoxamine-5′-phosphate (PMP) was released more slowly into the bulk, indicating that the mutation also enhances their PMP binding capacity. The crystal structure of PfTA, elucidated in this work, revealed a tetrameric arrangement with the PLP binding sites near the subunit interface. In this case, the V129N mutation had a negligible effect on PLP-binding, but it reduced its temperature stability possibly destabilizing the quaternary structure.

## Introduction

Pyridoxal 5′-phosphate (PLP) is the biologically active form of vitamin B_6_ and plays a key role in the enzymatic mechanism of a variety of biochemical reactions (Percudani and Peracchi, 2003; Eliot and Kirsch, 2004). PLP-dependent enzymes were initially classified in five different fold types, which were originally used to explain their different reaction specificities, but after further analysis, PLP-dependent enzymes are now classified in seven different major groups (Dunathan, 1966; Grishin et al., 1995; Percudani and Peracchi, 2009).

**Figure 1:**
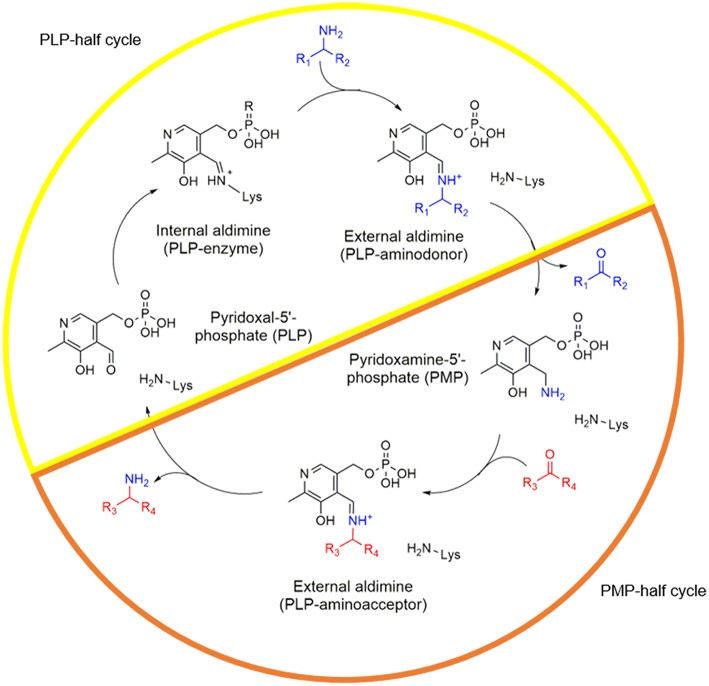
Catalytic cycle of TAs. The PLP half-cycle is depicted with a yellow half circle while the PMP-half life cycle with an orange half circle. The amino donor is colored blue and the amino acceptor molecule in red.

One of the most important groups among PLP-dependent enzymes are transaminases (TAs). These enzymes catalyze the transfer of an amino group from an amino donor to an amino acceptor. In short, the reaction starts with a holo-enzyme complex where PLP is initially bound to the enzyme through an imine bond with the ε-NH_2_ of the catalytic lysine (internal aldimine). This complex then reacts with the amine donor forming an external aldimine that after hydrolysis gives rise to pyridoxamine 5′-phosphate (PMP) and the deaminated product. If PMP remains at the active site, it readily interacts with the amine acceptor to form the aminated product and then returns to the holo-form of the enzyme (PLP-bound) to start a new catalytic cycle (Figure 1).

In recent years, TAs have attracted interest for the production of high value chemicals (Höhne and Bornscheuer, 2009; Kohls et al., 2014; Guo and Berglund, 2017). Broadly speaking, TAs can be divided into α- or ω-transaminases (ωTAs), depending on the position of the amino group transferred with respect to the carboxyl group of the substrate (Mathew and Yun, 2012). ωTAs which belong to the aminotransferase III family (EC 2.6.1.X, PF00202), offer a greener, safer and scalable approach for the synthesis of several pharmaceutical building blocks. Despite numerous efforts made for studying and optimizing TAs, only two variants have been implemented in industrial processes: ATA117, an *R-*selective transaminase engineered to be used in the industrial production of sitagliptin (Savile et al., 2010) and an *S-*selective transaminase from *Arthobacter citreus* for the synthesis of aminotetralin and the production of acetophenone (Martin et al., 2007). Major drawbacks for the industrial implementation of transaminases are related to their catalytic activity, and more importantly to their unfavorable operational stability.

The low operational stability of transaminases in amination reactions is often explained by the presence of high substrate concentration, the high amine donor to acceptor ratios, with the requirement for co-solvents to increase solubility, as well as PLP requirements to maintain the catalytic cycle. Hence, enzyme inactivation is partially explained by cofactor loss during prolonged bioconversions of large amount of substrate (Yun et al., 2005; Chen et al., 2018b). This phenomenon compromises both the activity and stability of transaminases since PLP and its aminated version, PMP, must remain in the active site to complete the catalytic cycle (Chen et al., 2018a). In applied biocatalysis the addition of exogenous PLP increases the operational stability under process intensification conditions, but the price of PLP limits the cost efficiency of the transaminase-based bioprocess at large scale. Interestingly, different TAs from different sources have varied affinity toward PLP as demonstrated by different requirements in terms of exogenous supply of cofactor (Mathew and Yun, 2012). Our group reported that ωTA from *Halomonas elongata* exhibited a 1.5 times higher total turnover number (TTN) of PLP per molecule of enzyme than its *Chromobacterium violaceum* homolog (Benítez-Mateos et al., 2018); this suggests that the former ωTA binds the cofactor more strongly than the latter. Protein engineering, by semi-rational mutagenesis, has been applied successfully to develop more robust transaminase-based biocatalysts to be used in industry (DiSanto et al., 2007; Savile et al., 2010; Börner et al., 2017a), but studies specifically targeting the optimization of PLP binding are scarce. Often, the optimization for substrate binding and activity causes a lower affinity for PLP. For example, the transaminase mediated synthesis of sitagliptin demands 4 times higher PLP supply in the scaled-up process.

**Figure 2:**
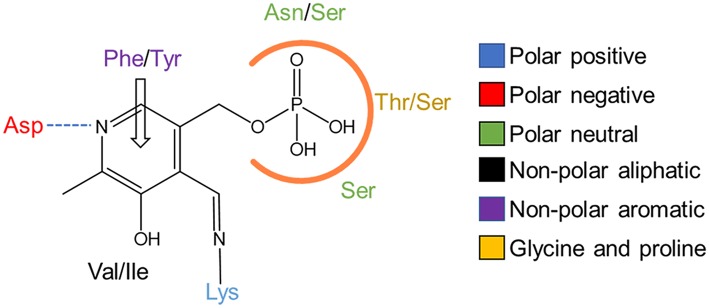
PLP binding in TAs. 2D representation of the key interactions in the PLP binding pocket. Hydrogen bonds are shown as a dashed blue line, π-π stacking as an empty arrow and the phosphate binding cup is marked with an orange line. Amino acids are colored as shown in the legend.

Depending on the origin of the ωTA, PLP binding displays different involvement in favoring energetic protein conformation to efficiently perform the catalysis. For example, PLP binding has a negligible contribution to stabilizing the active conformation of the dimeric CvTA yet the cofactor occupies both active sites (Humble et al., 2012). On the contrary, the active conformation of a tetrameric enzyme from *Pseudomonas* sp. becomes highly stabilized by PLP binding, but the structural data suggest a cooperative binding of PLP since it only occupies one out of the four active sites per turnover (Sayer et al., 2013). Despite the different structural consequences induced by PLP binding on ωTAs, the cofactor interactions within the active site are very similar (Figure 2). ωTAs demand a protonated cofactor to accomplish the catalytic cycle. This protonation state relies on an aspartate residue which ionically interacts with the pyridinium nitrogen (Eliot and Kirsch, 2004). In addition, PLP is tightly bound to the catalytic site through π-π stacking interactions and hydrogen bonds established between the aromatic ring and the phosphate group, respectively (Denesyuk et al., 2003).

In the present study, we intend to better understand the molecular interactions that govern PLP binding in three industrially relevant ωTAs, namely from *Halomonas elongata* (HeTA) (Cerioli et al., 2015), *Chromobacterium violaceum* (CvTA) (Kaulmann et al., 2007) and *Pseudomonas fluorescens* (PfTA) (Ito et al., 2011; Mutti et al., 2012). Careful analysis of the multi-sequence and structural alignments reveal that HeTA presents an Asn suitably positioned to interact with either the pyridinium nitrogen or with an Asp (Asp255) that keeps PLP protonated. In the equivalent position, the two other studied enzymes (CvTA and PfTA) have a valine instead. By constructing three different single mutants (HeTA-N120V, CvTA-V124N and PfTA-V129N), we gathered experimental evidences that supports the role of this Asn in PLP binding. This residue appears to enhance a stable interaction with the cofactor explaining the higher operational stability of HeTA under high substrate and limited PLP concentrations compared to CvTA and PfTA (Benítez-Mateos et al., 2018).

## Materials and Methods

All chemical reagents, unless stated otherwise, were purchased as analytical grade from Sigma-Aldrich, U.K., Acros Organics or Thermo Fischer Scientific U.K.

### Crystallization, Data Collection, and Structure Determination of PfTA

A full length PfTA synthetic construct, cloned in pET-28b vector (see Supplementary Information), was expressed in the *Escherichia coli* BL21 codon plus strain cultured at 37°C in LB medium. Expression was induced by addition of 1-thio-β-D-galactopyranoside at 0.5 mM and cells harvested after 3 h. Cells were lysed by sonication in 45 mM phosphate, pH 7.2, 0.9 M NaCl, 0.09 mM PLP, 9 mM imidazole, 10% (v/v) glycerol. Samples were purified on a HisTrap HP chelating column (GE Healthcare) pre-charged with nickel sulfate. Further purification was carried out by size-exclusion chromatography using a Superdex 200 HiLoad 16/60 column (GE Healthcare) pre-equilibrated with 50 mM HEPES, pH 7.5, 300 mM NaCl, 0.1 mM PLP, 1% glycerol. Sitting drop vapor diffusion crystallization trials were carried out at 20°C and the best hits were obtained in conditions G1 and G6 from the LMB screen (Molecular Dimensions), which were further optimized by varying protein and precipitant concentrations. Single crystals grew in under a week; the best condition being 6 mg/mL protein (in 50 mM HEPES, pH 7.5, 300 mM NaCl, 0.1 mM PLP, 1% glycerol, 20 mM DTT) mixed 1:1 0.1 M sodium citrate, pH 5.6, 12% w/v PEG 6000, 0.1 M lithium sulfate. Crystals were flash cooled after soaking in cryoprotectant solution containing six parts precipitant mixture with four parts glycerol. Diffraction data for these crystals were collected on the I24 beamline at the Diamond Light Source and the structure was solved by molecular replacement using coordinates from the uncultivated *Pseudomonas sp*. transaminase (PDB: 5LHA) as a search model with PHASER.

### Model Building, Refinement, and Validation

Model building was conducted using COOT (35), and refinement using REFMAC5 (Murshudov et al., 2011). Data collection and refinement statistics are summarized in supporting Supplementary Table 1. The final model was validated using Molprobity and deposited into the Protein Data Bank (PDB: 6S54).

The asymmetric unit contained four molecules in a tetrameric arrangement, with an average interface of 5428.1 Å^2^ between two subunits within a dimer and 1071.4 Å^2^ between a subunit within one dimer and its nearest neighbor in the opposite dimer using the PISA server (Krissinel, 2015). Clear electron density for PLP linkage to K293 was observed in two chains (A and B), whereas the K293 side chain was less well defined in chains C and D, which may reflect a lower occupancy of the cofactor and/or a portion of the cofactor non-covalently bound to the enzyme. The latter two chains displayed higher B-factors overall. Figures were prepared using VMD (Humphrey et al., 1996) or Pymol (L DeLano, 2002).

### Computational Analyses

3-dimensional structure analyses were done using the crystal structures of HeTA (PDB: 6GWI) (Planchestainer et al., 2019), CvTA (PDB: 4A6T), and for PfTA (PDB: 6S54). Residue interaction analysis was performed using Cytoscape (Shannon et al., 2003) in combination with StructureViz and RINanalyzer.

### MSA and Conservation Scores

For aspartate aminotransferases, multiple sequence alignment was done retrieving all available sequences form the oTAED (Buß et al., 2018) annotated to be class III transaminases, corresponding to Pfam PF00202 (24998 sequences in total). Multiple sequence alignment was performed using ClustalW with two iterations. The PLP binding site was analyzed using HeTA, CvTA, and PfTA crystal structures.

For D-alanine aminotransferases (PF01063) and serine hydroxymethyltransferase (PF00464), the alignment was retrieved from the Pfam database with a total number of 11,689 sequences and 7,460, respectively. The amino acid conservation in each position was analyzed using Bioedit and the graphical visualization was performed with WebLogo (Crooks et al., 2004). Positions corresponding to the PLP binding site were visualized and analyzed using DAAT from Bacillus sp. YM-1 crystal structure (PDB: 3DAA) as a model (Peisach et al., 1998) and the serine hydromethyltransferase from *Bacillus stearothermophilus* (PDB: 1KKP) (Trivedi et al., 2002).

### Protein Expression and Purification

HeTA and CvTA and its mutants were expressed in *E. coli* BL21 (DE3) Star in ZYP autoinduction media at 37°C. One single colony was used for inoculation and left to grow for 20 h before collection. The pellet was collected by centrifugation for 20 min at 4,415 x g and then resuspended in 50 mM phosphate buffer, 0.3 mM NaCl pH8, sonicated for 10 min (in 30 s pulses) and centrifuged 1 h at 22,769 × g to remove the cell debris. The proteins were purified by IMAC chromatography (GE Healthcare, Histrap 5 mL FF) and dialyzed against 50 mM phosphate buffer pH8 overnight (Kaulmann et al., 2007; Cerioli et al., 2015).

For PfTA and its mutant, the proteins were expressed in *E. coli* BL21 (DE3) Star in LB media at 37°C until it reached an optical density at 600 nm of 0.5–0.7; 0.01 mM of IPTG was added and the cultures left to grow for 16 h at 21°C. The pellet was collected by centrifugation for 20 min at 4,415 × g and then resuspended in 50 mM phosphate buffer, 0.1 M NaCl pH7, sonicated and the cell debris removed by centrifugation for 1 h at 22,769 × g. The protein was purified by IMAC chromatography and dialyzed against 50 mM phosphate buffer pH7, 0.1 M NaCl overnight (Mutti et al., 2012).

Protein concentration was calculated using an extinction coefficient of 62,840 M^−1^ cm^−1^ and a molecular weight of 54.4 kDa for the monomer for HeTA, 81,735 M^−1^ cm^−1^ and 55.2 kDa for CvTA and 68,340 M^−1^ cm^−1^ and 52.5 kDa for PfTA, calculated using ProtParam online resource (Gasteiger et al., 2005).

### Apo and Holo-Form Calculations

The ratio of absorbance Abs415/Abs280 was used to assess the percentage of holo-form after purification, with no external PLP. For HeTA, CvTA and its mutants the concentration range was from 5 to 7 mg/mL while for PfTA and its mutant concentration was 0.7–1.2 mg/mL. The values were compared with the same sample after incubation for 12 h with 5 mM PLP at 4°C and 1 h at 37°C after elimination of unbound or free PLP using gel filtration (PD-10 column purchased from GE Healthcare). The fractions containing the protein were collected and the ratio Abs415/Abs280 measured fixed as being the 100% holo-form.

### Enzyme Activity Assay

The standard assay to measure activity was performed using 2.5 mM pyruvate, 2.5 mM S-MBA and 0.1 mM PLP in phosphate buffer pH8. The reaction was performed indistinctly in 1 mL or 300 μL final volume and monitored at 245 nm on an Epoch 2 microplate reader, and velocities were calculated using an extinction coefficient of 12,600 M^−1^ cm^−1^ for acetophenone. A unit is defined as the amount of enzyme capable of converting 1 μmol of substrate per minute.

### Cofactor Release Study

To a solution of 0.5 mg/mL of the enzyme, 50 mM benzylamine and 0.5 mM of PLP were added. The absorbance was measured every 5 min for 4 h at 395 nm to monitor the depletion of PLP and at 325 nm to measure the formation of PMP at 25°C using an Epoch 2 microplate reader. The absorbance values were normalized by subtracting the minimum absorbance and dividing by the maximum difference, so all values ranged between 1 and 0.

### Temperature Stability Assays

The different proteins at a concentration of 1 mg/mL for CvTA and HeTA and 0.1 mg/mL of PfTA in their apo-form were incubated for 30 min at different temperatures ranging from 25 to 65 degrees in 50 mM phosphate buffer pH8 without external PLP. After the incubation, samples were centrifuged to eliminate the precipitated protein and were left in ice for another 30 min. The activity was then tested following the formation of acetophenone at 245 nm with the standard assay.

### Melting Temperature and Dissociation Constant Calculations

Melting temperature (T_1/2_) values were calculated using differential scanning fluorimetry (DSF) using a BioRad C1000™ Thermal cycler and a CFX™ 96 Real Time System measuring the fluorescence in the range of the SYPRO Orange dye. The sample consisted of 1 mg/mL apo-form protein, 15x Sypro Orange with or without 0.25 mM PLP in 50 mM phosphate buffer pH8. The melting temperatures were calculated fitting the data to a two-state Boltzmann function using GraphPad Prism™.

*K*_*d*_ values of PLP for the apo form of each protein were calculated using different concentration of PLP (1–250 μM) in 50 mM phosphate buffer pH8. Each sample was prepared with the desired amount of PLP in the same conditions as before. A single-site binding was then used to estimate the *K*_*d*_ from the T_1/2_ values obtained using GraphPad Prism™.

## Results

### PfTA Crystal Structure

**Figure 3:**
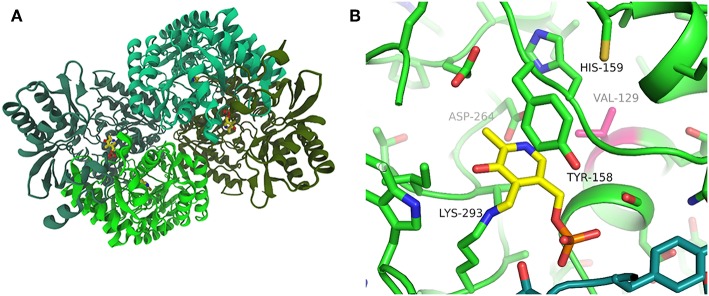
Structure of PfTA: **(A)** Overall structure of PfTA in cartoon representation and PLP shown in yellow sticks highlighting the tetrameric arrangement (subunits in different shades of green) **(B)**. Close-up view of the PLP (yellow) binding site with key residues labeled and Val129 highlighted in magenta. Residues from the two subunits forming the PLP binding site are colored in green and teal, respectively.

In order to compare the three enzymes at the molecular level we crystallized PfTA in the presence of the cofactor, PLP. Data from crystals grown in a condition with PEG6000 as the precipitant diffracted to 2.2 Å resolution and the structure was solved by molecular replacement (Supplementary Table 1). The crystal structure showed a tetrameric arrangement of PfTA with the cofactor positioned at the interface of two subunits and PLP linked to K293 (Figures 3A,B). V129 is well-defined and in proximity to D264, which is within hydrogen bonding distance to H159 and the pyridinium nitrogen. The PfTA structure is most closely related to structures of a TA from an uncultivated *Pseudomonas* species (PDB: 5LH9, 5LHA; Börner et al., 2017a) with which it shares 49% sequence identity. This enzyme also has a valine at the equivalent position to V129 and displays a similar overall tetrameric structure. The structures can be superimposed with an RMSD of corresponding residues of 1.0 Å over 443 residues (Supplementary Figure 3). On the other hand, HeTA (PDB: 6GWI) has a sequence identity of 40% and CvTA (PDB: 4A6T) of 36% with RMSDs of 1.5 and 1.8 Å when superimposed, respectively. We next performed more detailed analysis of the three enzymes under investigation.

### Residue Interaction Analysis and Conservation

**Figure 4:**
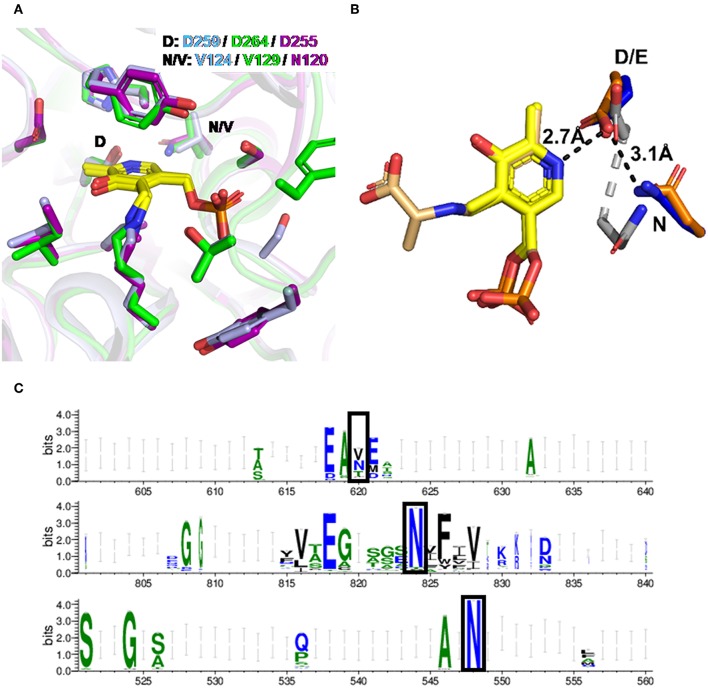
PLP binding in HeTA. **(A)** Superposition of the PLP binding site for HeTA in purple (PDB: 6GWI), CvTA in light blue (PDB: 4A6T), and PfTA in green (PDB: 6S54). PLP is depicted in yellow. The aspartate interacting with the pyridinium nitrogen and the position of interest are labeled. **(B)** Detail of the interaction of the pyridinium nitrogen with the aspartic or glutamic residue for HeTA (blue), DAAT in gray (PDB: 3DAA), and SHMT in orange (PDB: 1KKP). PLP is depicted in yellow and the serine bound-PLP for the SHMT in light yellow. Distances in angstroms are shown for HeTA with black lines. **(C)** Amino acid frequency in the homologous position for TAs class III (top), DAAT (middle), and SHMT (bottom).

To understand the molecular basis of the differences between HeTA, CvTA, and PfTA, we compared their sequences (Supplementary Figure 1) and available 3D structures of aminotransferase family III. The 3D-structures of the three studied enzymes were superimposed (Supplementary Figure 2) and all three PLP-binding sites are formed by almost identical residues in equivalent positions, matching the expected geometry previously described for ω-transaminases (Figure 4A) (Łyskowski et al., 2014; Guidi et al., 2018). Nonetheless, a minor but potentially relevant difference in the PLP-binding pocket was found through residue interaction analysis (RIN) (Supplementary Figure 3). Interestingly, an Asn at position 120 (N120) of HeTA caught our attention as possibly having an important role in the interaction network with PLP. This residue is positioned at an α-helix near the interface between the two monomers. In contrast, the corresponding residue in PfTA and CvTA is a valine with no predicted interaction with PLP. The presence of such an Asn in this position might explain the stronger binding of PLP in the HeTA active site.

Remarkably, within the same fold type, serine hydroxymethyltransferases (SHMT, EC 2.1.2.1) or even in fold type IV, D-aminoacid aminotransferases (DAAT, EC 2.6.1.21), have a highly conserved Asn in this position (Figures 4B,C). These two groups of enzymes also catalyze PLP dependent reactions in a similar manner as TAs and, although they have a different architecture, the main interactions forming the PLP-binding pocket are conserved, especially those that rely on the catalytic lysine, the “phosphate-cup” and the negatively charged amino acid that interacts with the pyridinium nitrogen (Supplementary Figure 4).

In class III aminotransferases, multi-sequence alignment of all entries annotated as such indicated that Asn is not especially conserved in that position. In fact, Asn and Val are similarly abundant, followed by Thr (Figure 4C).

Inspection of the superimposed structures shows that the N120 in HeTA belongs to a second PLP interaction shell that faces the carboxyl group of the vicinal D255 residue (first PLP interaction shell), known to maintain the cofactor protonated (Eliot and Kirsch, 2004). The distances between D255 and the pyridinium nitrogen in HeTA is 2.7 Å, whereas the amide-nitrogen of N120 is positioned at 3.1 Å from the carboxylic group of D255 (Figure 4B). The vicinity of these residues suggests that N120 may form a hydrogen bonding interaction with the carboxyl group of D255. This interaction may help to effectively position D255, favoring its electrostatic interaction with the nitrogen from the pyridinium group of the cofactor and thus, strengthening the enzyme-PLP supramolecular binding, which would avoid the premature release of both PLP and PMP during the catalytic cycle. The role of such an acidic amino acid on PLP binding has been demonstrated by mutational studies where TA variants lacking an Asp or Glu at that position displayed a significantly reduced catalytic efficiency (Wu et al., 2011; Son and Kim, 2016).

To assess the role of N120 in cofactor binding, three single mutants of HeTA, CvTA, and PfTA were constructed. HeTA-N120V aims to disrupt the postulated interaction between N120 and D255, while CvTA-V124N and PfTA-V129N were designed to mimic the interaction network found in HeTA and were consequently hypothesized to increase the PLP binding affinity. All mutants were successfully created, expressed and purified following the same protocol as for the WT enzymes.

### Functional Characterization of ωTAs Single Mutants

UV-Vis spectroscopy was utilized to quantify the holoenzyme fraction obtained after the overexpression of native and single-mutants of HeTA, CvTA and PfTA. The internal aldimine formed by PLP covalently bound to the catalytic lysine shows an absorption maximum at 415 nm, unlike the free PLP, whose maximum is at 390 nm (Chen et al., 2018a). Based on these spectral differences, we quantified the fraction of purified ωTA bound to PLP (holo-form) by comparing 415 and 280 nm absorbance values (Supplementary Figure 5). To this aim, we quantified the holo-form in freshly purified enzymes (both wild-type and mutants) incubated or non-incubated with exogenous PLP (Chen et al., 2018a). The former samples were considered as the maximum achievable fraction of holo-form (100%) where all enzymes contain a covalently linked PLP molecule per subunit. To estimate the occupancy of PLP in freshly produced enzymes, the absorbance at 415 nm was normalized by the concentration of protein in mM. The values for the sample purified without PLP were compared with maximum theoretical occupancy (Table 1).

**Table 1:** Holo-form percentage and K_d_ values for PLP.

		**Holo-form (%)**	**Occupancy^*a*^ (PLP:enzyme ratio)**	***K_*d*_* (μM)**
HeTA	WT	69 ± 2	1.39 ± 0.04	12.4 ± 3.6
	N120V	42 ± 9	1.19 ± 0.02	30.3 ± 4.1
CvTA	WT	16 ± 2	0.33 ± 0.01	59.0 ± 15.8
	V124N	13 ± 1	0.22 ± 0.01	16.4 ± 5.6
PfTA	WT	39 ± 8	1.57 ± 0.27	14.1 ± 2.3
	V129N	37 ± 9	1.64 ± 0.04	14.8 ± 2.5

*The holo-form percentage of each protein was calculated as stated in the methods*.

*K_d_ values for PLP are presented in μM. Values were obtained fitting to a 1-site binding the values of T_1/2_ obtained at various concentrations of PLP*.

a *Maximum occupancy is estimated as 2 for the two dimeric transaminases, HeTA and CvTA, and 4 for PfTA*.

WT-HeTA exhibited the highest holo-form fraction (~70%) and occupancy values (1.39) of all TAs herein studied. On the other hand, PfTA presented a 40% initial holo-form and an occupancy value of ~1.6 while only 16% of CvTA appeared to have PLP bound, with an occupancy value of 0.3, which suggests that most CvTA molecules lack PLP. Interestingly, the HeTA-N120V mutant had nearly a 20% decrease of the holo-form which translates to a lower occupancy but introducing an Asn in CvTA-V124N and PfTA-V129N resulted in enzyme preparations with similar fraction of initial holo-form and occupancy as their native counterparts.

To further investigate the affinity of these enzymes toward PLP, the PLP dissociation constant (*K*_*d*_) was calculated (Table 1) from the midpoint denaturation point (T_1/2_) at different concentrations of PLP. *K*_*d*_ is an equilibrium constant used to define the binding affinity of an enzyme for a ligand, in this case PLP. A lower value for *K*_*d*_ indicates a higher binding affinity of the enzyme for PLP. HeTA exhibited a similar *K*_*d*_ value as the tetrameric PfTA but a 5 times lower value than WT-CvTA. When PLP affinity was determined for the mutants, we observed that replacing N120 by a valine in HeTA decreased the affinity toward the cofactor by 3-fold. Remarkably, CvTA-V124N had a significant increase in the PLP binding capacity, with nearly 3-fold lower *K*_*d*_ than the wild-type enzyme. The CvTA-V124N new variant showed a *K*_*d*_ comparable to that of WT-HeTA and WT-PfTA. These results suggest that N120 of HeTA reinforces the interaction network of the acidic residues that anchors the pyridinium ion of PLP (Figure 4A). Surprisingly, the introduction of N into the corresponding position in PfTA (V129N variant) only negligibly affected the PLP binding affinity. The engineering of CvTA to CvTA-V124N indeed improves the enzyme affinity toward the cofactor, but such improvement was not observed for PfTA, likely because the contribution of that interaction is less relevant in a tetrameric TA.

**Figure 5:**
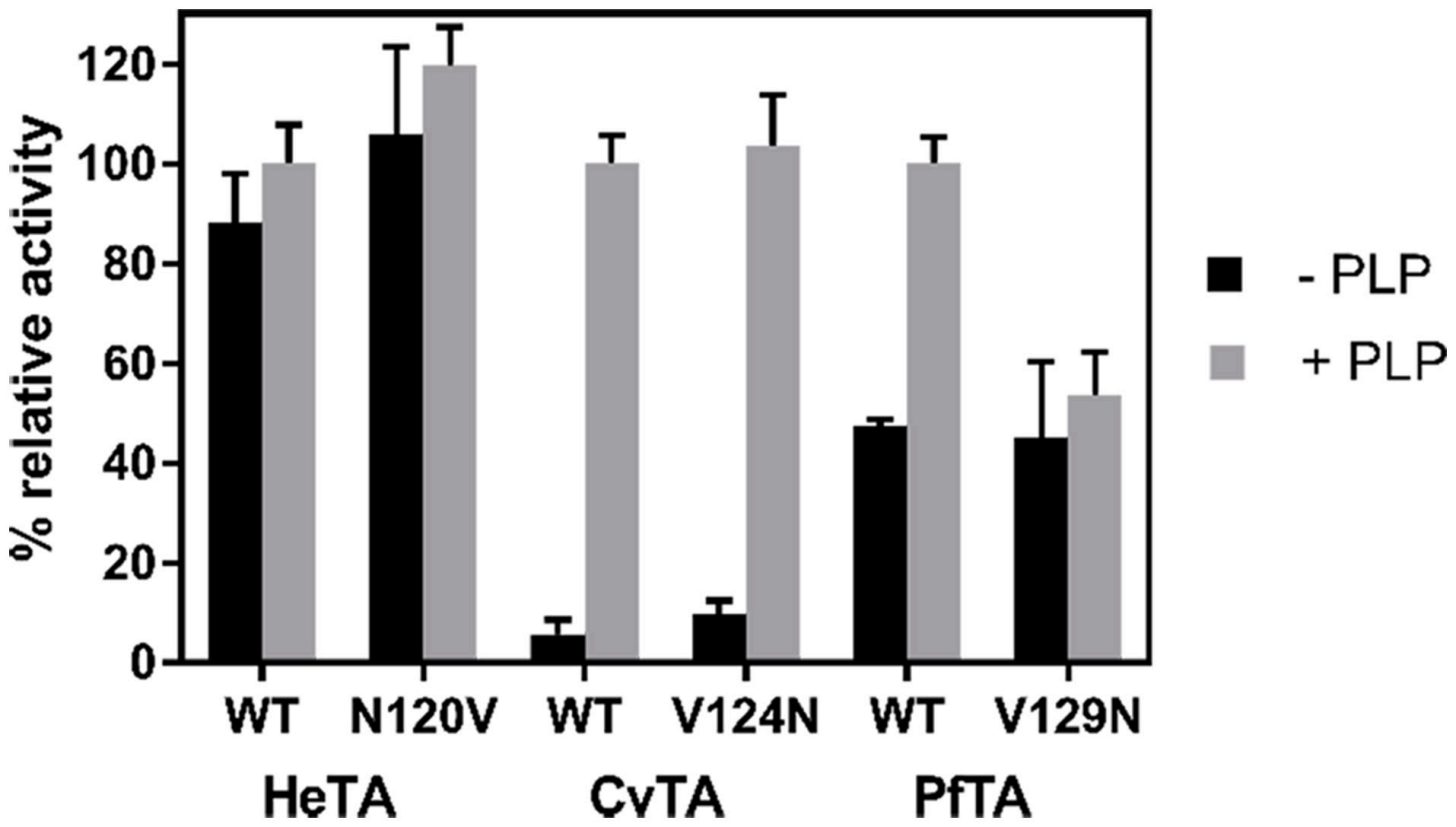
Activity of ωTAs in the presence and in absence of PLP. Values correspond to the mean between three replicates from two different protein batches with the standard deviation. The activity was assessed using the standard assay with (gray bar) and without (black bar) PLP. 100% of activity corresponds to the activity of each WT-TA after incubation with PLP.

Moreover, the effect of PLP affinity on the functionality of the enzyme was assessed by measuring the specific activity of all native and single-mutated variants with and without exogenous cofactor (Figure 5). For both native and N120V HeTA variants, the exogenous addition of PLP marginally affected the specific activity, implying that HeTA operates at its maximum capacity when more than half of the active sites are occupied by PLP. However, the saturation of CvTA active sites with PLP significantly increased its specific activity for both native and mutated (V124N) variants. Unexpectedly, in the case of PfTA, we observed that the WT variant displayed a 2-fold increase in its specific activity under saturating PLP conditions like CvTA, but the activity of the variant PfTA-V129N was insensitive to the exogenous addition of PLP.

From these results, we conclude that HeTA activity is less dependent on exogenous PLP than native PfTA and CvTA. HeTA-N120V showed a similar specific activity as the native enzyme, despite the mutation decreasing the PLP-binding affinity. These data suggest that the mutation does not affect the catalytic efficiency of the enzyme but may promote a premature dissociation of the cofactor that can negatively impact on the biocatalyst stability and recyclability. Therefore, N120 may promote tighter binding of PLP and PMP within HeTA active site. The role of this Asn on the PLP binding is also supported by the fact that CvTA-V124N was slightly more active than the native variant under PLP limiting conditions. The activities of both native and mutated enzymes were equal when saturated with the cofactor. However, the same mutation on PfTA made that variant insensitive to the cofactor concentration, unlike the native enzyme which increased its activity under PLP saturating conditions. While the introduction of an Asn into CvTA makes an enzyme with similar PLP affinity as the native HeTA, the same mutation into PfTA achieves the PLP insensitivity observed in HeTA.

### PMP Release in the Absence of the Amino Acceptor

**Figure 6:**
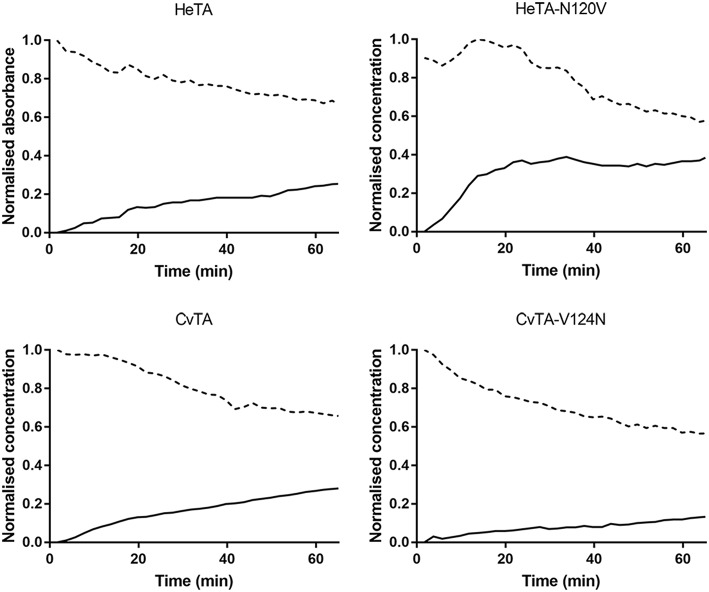
Time-courses of PMP formation during the first half of the transamination reaction. From a reaction containing 0.5 mM enzyme, 50 mM benzylamine and 0.5 mM PLP, the formation of PMP (solid line) was measured by following the increase of absorbance at 325 nm while PLP (dashed line) was monitored at 390 nm. Values of absorbance were normalized.

To further comprehend the effect of the Asn residue, the first half reaction of the transaminase catalytic cycle was studied by measuring the absorbance at 390 nm for PLP and 325 nm for PMP in absence of amine acceptor (Börner et al., 2016). Using these values, the depletion of PLP and formation of PMP in the bulk were monitored for 4 h (Supplementary Figure 6). The initial release of cofactor is shown in Figure 6.

In the assayed conditions, PLP reacts with the amine donor to yield PMP which is only stabilized through non-covalent binding. Therefore, a higher affinity of the aminated cofactor corresponds to a lower rate of PMP accumulation in the bulk. Likewise, a high rate for PMP formation indicates that the active site of the enzyme is exchanging more rapidly the PMP molecules formed during the first half of the catalytic cycle, leaving it accessible for other exogenous PLP molecules to start a new first-half cycle. Since the carbonyl acceptor is missing, the catalytic cycle cannot be completed, forcing the accumulation of PMP in (and depletion of PLP from) the reaction media.

HeTA-N120V produces PMP ~1.5 times faster than wild type HeTA over the first 60 min, with a much steeper increase in the formation during the first 20 min (Figure 6). Similarly, CvTA aminates PLP ~2.15 times faster than CvTA-N120V. These results indicate that PMP is less tightly bound and more easily leaves the active sites in the variants lacking the targeted Asn. In summary, in the absence of a stabilizing Asn residue (HeTA- N120V and WT-CvTA), the PMP is more rapidly released to the reaction bulk.

### Thermal Stability of Transaminases Under PLP Limiting Conditions

**Figure 7:**
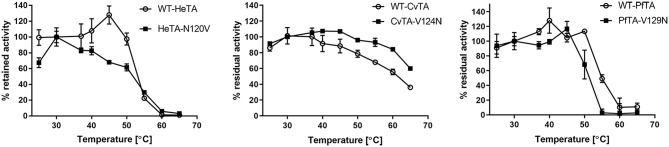
Temperature stability assays. The activity of the WT and the mutants for the three transaminases after incubation at different temperatures for 30 min with no external PLP is presented. 100% is defined as the activity at 30°C.

To investigate the implications of this mutation on the stability of the three ω-transaminases herein studied, HeTA, CvTA, and PfTA and their corresponding single mutants were incubated for 30 min at different temperatures without external addition of PLP (Figure 7). The activity of the samples was then measured and compared with the value obtained for the same sample at 30°C. The thermal inactivation was performed with the enzymes purified without exogenous PLP to evaluate the resistance of the PLP-enzyme interaction.

Expectedly, HeTA-N120V was significantly less stable than its native counterpart, due to the lower affinity for PLP and CvTA-V124N was more stable than the WT-CvTA, indicating that the mutation stabilizes the protein because of stronger PLP binding. However, the mutation negligibly affected PfTA's thermal stability below 50°C, but at higher temperatures the residual activity of PfTA-V129N was noticeably lower than for the wild type (Figure 7C).

The data obtained from the thermal incubation assays might reflect conformational changes that drive the loss of enzyme activity. The structural rearrangements may be related to the dissociation of the PLP-enzyme complex, leaving a catalytically inactive form, rather than the protein unfolding. In the HeTA-N120V variant, the lack of interaction between N120 and D255 seems to weaken the PLP-enzyme interaction, promoting a premature release of the cofactor that diminishes the protein's conformational stability, causing its inactivation. Aligned with these results, the introduction of an Asn in the homologous position of CvTA increases its thermal stability, likely due to the reinforcement of the enzyme-PLP complex. These insights agree with inactivation studies that demonstrate that this enzyme was more stable when the incubation was performed in presence of an excess of PLP (Chen et al., 2018a).

Unexpectedly, PfTA showed the opposite behavior since the variant V129N was less thermally stable than the native enzyme. As it assembles as a tetramer, the mutation may negatively affect the quaternary structure as it does not affect the stabilization of the enzyme-PLP complex. PfTA has 49% sequence identity to a tetrameric ωTA from a uncultivated *Pseudomonas sp*. (PsTA) whose crystal structure has been resolved (Börner et al., 2017b). In both cases, the corresponding Val residues (V129 in PfTA and V119 in PsTA) are part of an alpha helix located near to the oligomerization interface; between two monomers and near to the dimer-dimer interaction. Therefore, the replacement of that Val by an Asn may disrupt some crucial hydrophobic interactions required for the assembly of the quaternary structure needed for the enzyme stability, but also for the enzyme activity under PLP saturation conditions (Figure 4).

### Thermodynamic Unfolding of Transaminases

**Figure 8:**
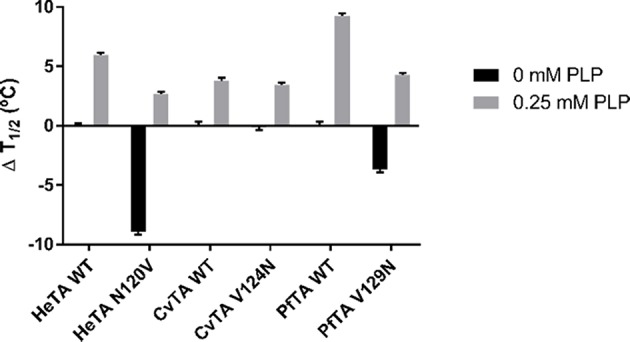
Thermal denaturation assays. ΔT_1/2_ values for HeTA, CvTA, and PfTA and its mutants is presented in the absence of exogenous PLP and with 0.25 mM PLP in the reaction buffer. The ΔT_1/2_ is measured compared to the WT variant for each protein. The T_1/2_ without PLP of the wild-type enzyme was considered to be 0 (T_1/2_ = 53.3°C for HeTA, 68.9°C for CvTA, and 62.9°C for PfTA).

Functional inactivation may be explained by either structural rearrangements, which decrease both substrate and cofactor affinities or by quaternary structure disassembly without the loss of folding of each subunit. However, both minor structural distortions caused by the cofactor release and the loss of quaternary structure speed up the protein unfolding as seen for other TAs (Humble et al., 2012; Börner et al., 2017b). To investigate whether lower affinities for PLP also promote protein unfolding, we performed thermodynamic denaturation studies to calculate the melting temperatures of the herein studied ωTAs and their mutants. The melting or half-life temperature (T_1/2_) corresponds to the temperature at which 50% of the population is unfolded, assuming a two-state transition state model. T_1/2_, values in the absence or presence of exogenous PLP, were calculated by differential scanning fluorometry using Sypro Orange dye (Figure 8) to assess the effect of the ligand.

Under limiting PLP conditions, the removal of N120 in HeTA shifted the T_1/2_ significantly, as HeTA-N120V showed a decrease of 9°C compared to the T_1/2_ of the native enzyme. Under PLP saturating conditions, the mutant T_1/2_ was only 3°C lower than the wild type. These results, in combination with the fact that the mutant presents a lower percentage of holo-enzyme (Table 1) and lower thermal stability (Figure 7A), supports the postulated role of the studied Asn in the PLP binding and the stability of TAs.

In the case of CvTA-V124N however, the mutation did not protect the enzyme from thermal denaturation in the absence of exogenous PLP, but both the mutant and the wild-type showed a 3°C increase in T_1/2_ under saturation conditions. In this case, lower PLP affinities do not trigger enzyme inactivation through unfolding but, more likely, through the premature dissociation of the cofactor (Figure 7B).

Like in the stability tests (Figure 7C), PfTA-V129N tends to unfold at a T_1/2_ value 3°C lower than WT-PfTA does, under limiting PLP conditions. A similar trend was found under PLP saturating conditions as T_1/2_ values of both native and mutant variants were higher. Nevertheless, the mutant unfolded at 5°C less than the native variant. Here the mutation has deleterious effect on the protein stability, which is more dramatic in the presence of exogenous PLP. In this case, the presence of the Asn seems to promote enzyme destabilization at both functional (Figure 7C) and structural levels (Figure 8).

## Discussion

The capacity of transaminases to bind PLP is linked to their operational stability. During the first half of the transaminase reaction cycle, PLP is aminated giving rise to PMP that is less tightly bound to the active site. Hence, the aminated cofactor can be more easily dissociated from the active site, turning the enzyme into its apo-form, which is more prone to inactivation, unfolding or even precipitation. HeTA, a dimeric transaminase from *Halomonas elongata*, exhibited higher operational stability than CvTA and PfTA, when used in continuous flow (Benítez-Mateos et al., 2018). Analysis of the PLP binding pockets in these three homologous transaminases has elicited key differences in their PLP-binding residue interaction network. The Asn residue located at position 120 in HeTA's primary sequence corresponds to a Val in the other two wildtypes ωTAs studied. Multi-sequence alignment of the aminotransferase III family showed that such Asn residue is not particularly conserved. In ωTAs, this position belongs to a secondary PLP interaction shell that strengthens the PLP-enzyme interaction. The poor conservation of this position amongst aminotransferase III family members may be due to divergent evolutionary pathways that can lead to enhanced PLP binding through alternative mechanisms. For example, ω-TAs can stabilize the secondary interaction network through capping of the PLP binding pocket with an adjacent subunit in tetramers or with hydrophobic clamps that fix flexible loops in dimers (Börner et al., 2017a,b). In HeTA though, the formation of a hydrogen bond between N120 and D255 is postulated here to optimize the ionization state of the carboxylic acid group, strengthening its ionic interaction with the pyridinium moiety of protonated PLP. Depending on the nature of the residues in the secondary shell, PLP affinity may vary amongst ωTAs. To demonstrate the role of this Asn, we constructed 3 different mutants: HeTA-N120V (designed to disrupt the hypothesized interaction) in HeTA, and CvTA-V124N and PfTA-V129N (designed to mimic the interaction predicted in HeTA).

After a substantial set of biochemical studies involving thermodynamic and kinetic tests, we have gathered sufficient evidence to demonstrate the influence of N120 on the binding of PLP. The *K*_*d*_ measurements clearly indicate that the presence of Asn has a significant impact on the cofactor affinity in the two dimeric structures. The *K*_*d*_ of HeTA-N120V for PLP is increased more than 2-fold with respect to the WT, and even more remarkably, the *K*_*d*_ of CvTA-V124N drops almost 3 times with respect to the WT, approaching the values of the WT-HeTA and WT-PfTA. For HeTA, this is further supported by the determined holo-enzyme fraction and PLP occupancy, while the CvTA-V124N mutant had no improvement in the PLP uptake during expression. Moreover, the removal of the interactions between the amide group of N120 and D255 of HeTA seemed to have a negligible effect on the transaminase activity although the cofactor affinity was diminished, which might ultimately have more implications regarding the stability of the biocatalysts. In the light of these results, the role of the targeted Asn seems to be related to the stability of the holo-enzyme (the active form) rather than to the catalytic efficiency of ωTAs.

In addition, in the dimeric enzymes, the presence of the studied Asn also minimized the extraction of PMP from the active site during the first half of the transamination cycle. Börner et al. (2017b) reported on how PMP-enzyme dissociation is one of the major contributors to the low operational stability of transaminases. In this case, the presence of the Asn in the proximity of the critical Asp residue seems to strengthen the non-covalent interaction with the pyridinium ion of the cofactor aromatic ring, hampering the loss of PMP and further stabilizing the protein-PMP complex needed to complete the second-half of the catalytic cycle.

A functional implication of lower PLP affinity amongst the TAs is reduced thermal stabilities. It has previously been reported that higher thermal stability in ωTAs correlates with higher PLP-binding affinity, since the bound cofactor reduces the overall protein flexibility, conferring it a higher tolerance to denaturation (Celej et al., 2003). HeTA-N120V was indeed inactivated at lower temperatures than WT-HeTA. Furthermore, the dissociation of PLP might trigger some structural rearrangements and facilitate protein unfolding as demonstrated by the lower T_1/2_ calculated upon thermal denaturation experiments. These T_1/2_ differences were mitigated under PLP saturating conditions, where cofactor dissociation was thermodynamically unfavored, avoiding the unfolding of the enzyme to some extent. Again, functional data obtained with the CvTA strongly supports these findings, since CvTA-V124N became a more thermostable variant than the wild-type. In the light of these results, the presence of Asn in CvTA seems to help it avoid inactivation through stronger cofactor binding rather than through protecting the protein from unfolding. In fact, no significant difference in T_1/2_ values was recorded despite their different *K*_*d*_ values for PLP.

The tetrameric PfTA, however, shows significantly different behavior. No apparent variation was observed between the WT and mutant for the *K*_*d*_of PLP dissociation, or in the formation of spontaneous holo-form and the PLP occupancy. Moreover, the thermal stability was not affected. Comparing the *K*_*d*_ values for PLP, it is notable that PfTA presents a similar affinity toward PLP as WT-HeTA does. This insight indicates that PLP binding in PfTA may be governed by the assembly of the quaternary structure rather than the amino acid present in the target position. The X-ray structure of PfTA herein solved, shows that the 4 PLP binding sites are near to the oligomerization interface, which may link the stability of the PLP-enzyme complex to the quaternary structure. These results support the findings of Börner et al. (2017b), who highlighted that tetrameric transaminases show higher operational stabilities due to the formation of stronger cofactor-enzyme complexes, minimizing the displacement of PMP. The locations of PLP binding sites might have remarkable implications in terms of enzymatic activity regulation. As for many other multimeric enzymes with active sites positioned near the oligomerization interfaces, we could speculate that PLP may exhibit a cooperative binding mode, in which a suitably high concentration of PLP is needed to trigger conformational changes which allow for occupancy of all sites to be achieved. This would explain the lower PLP occupancy values calculated for both variants of PfTA despite them having *K*_*d*_ values similar to WT-HeTA.

**Figure 9:**
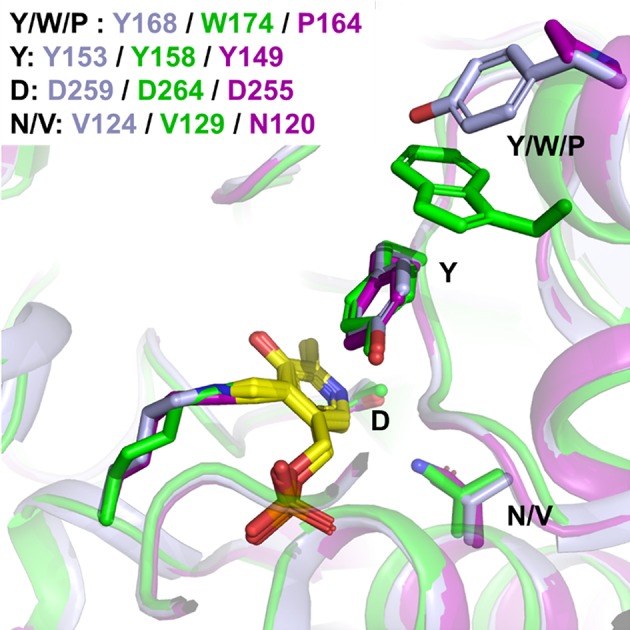
Detail of the PLP-ring motif. Detail of the flexible loop related to PLP binding. HeTA is shown in purple (PDB:6GWI), CvTA in light blue (PDB: 4A6T) and PfTA in green (PDB: 6S54). The residues forming the PLP-ring motif and the acid residues stabilizing the pyridinium ion.

On the other hand, it is accepted that cofactor binding relies on supramolecular π-stacking interactions between the cofactor aromatic ring and a PLP-ring motif, described for *Vibrio fluvialis* (Shin et al., 2018), and shared by CvTA (Humble et al., 2012) and *Pseudomonas sp*. For the three TAs herein studied, such interactions mainly rely on a conserved Tyr (Y149 in HeTA, Y153 in CvTA and Y158 in PfTA), although it seems that the most stable position of such Tyr changes upon PLP binding (Figure 9). CvTA possesses another Tyr (Y168) which could act as a staple, fixing the loop once the PLP is bound to the active site. A similar structural rearrangement has been observed for the TA from *Vibrio fluvialis* and from *Pseudomonas sp* (Börner et al., 2017b; Shin et al., 2018). Interestingly, in PfTA, an additional Trp residue (W174) seems to further stabilize the Y159 of the PLP-ring motif into establishing π-π stacking interactions with the cofactor pyridine ring. In this case, the binding loop is also constrained by the interaction with another subunit of the tetrameric structure (Supplementary Figure 7). Both factors (the quaternary structure that provides a more rigid environment typical of tetrameric TAs (Börner et al., 2017b), and the presence of the non-conserved tryptophan in close vicinity to the tyrosine) might contribute into explaining the high PLP binding affinity. Recently, mutational studies of the PLP-ring motif in a homologous ω-transaminase from *Pseudomonas sp*. were reported to hamper the dissociation of PMP and retain the quaternary structure during the catalytic cycle. Börner et al. (2017a) targeted the tyrosine clamp to construct more stable variants, specifically through stabilizing the enzyme-cofactor complex, overall increasing the enzyme-PMP complex stability.

HeTA, however, does not present any hydrophobic residue or additional subunits to lock the position of the Y153 in the π-π stacking interaction, and yet despite that, the *K*_*d*_ value was similar to PfTA. In HeTA, the Trp of PfTA is replaced by a proline that hardly interacts with Y149. Therefore, the stability of the PLP binding in HeTA appears to be better justified by the hydrogen bonding between N120 and D255, as supported by the thermodynamic analysis of PLP binding and proved by the removal of this residue, which produces a HeTA variant with significantly less affinity toward the cofactor. The fact that both the dimeric HeTA and the tetrameric PfTA have a similar affinity for PLP clearly indicates that HeTA must possess some uncommon traits in the PLP interaction network which also are key for retaining the non-covalently bound PMP during the catalytic cycle. Hence, the extraction of PMP, and the consequent loss of enzyme structural stability, is minimized through hydrogen bonding more than by dynamic hydrophobic interactions, as described before for other well-studied ωTAs (Börner et al., 2017a,b).

In summary, we have here identified an Asn that is neither part of the phosphate cup, nor of the PLP-ring bonding motif, but which clearly plays a key role in PLP-binding. Structural information confirms that this residue is suitably positioned to establish a hydrogen bond with a fundamental and conserved acid residue (Asp/Glu) that interacts with the pyridinium moiety of PLP bound in the enzyme active site. The presence of an Asn in that position -either native or engineered- enhances the enzyme thermal stability under PLP limiting conditions, and also the biocatalyst operational stability by causing a higher affinity toward PLP and by minimizing PMP release during the first half of the transamination catalytic cycle. To date, no previous reports have pointed to the role of this asparagine in the PLP binding of aminotransferases. Yet, targeting that position, we have achieved an enhancement in PLP affinity comparable to previous attempts made with other PLP-dependent enzymes such as lysine decarboxylase, rationally engineered through the addition of disulfide bonds to achieve a 3-fold decrease in the *K*_*d*_ for PLP (Sagong and Kim, 2017).

Therefore, the identification of this position has opened up a new pathway for engineering dimeric TAs, for reinforcing the cofactor interactions to ultimately achieve more robust biocatalysts with higher potential for industrial applications. However, the role of that Asn in PLP binding is less relevant for tetrameric TAs, where the stability of the quaternary structure dominates the cofactor binding and consequently the stability of the reactive cofactor-enzyme complexes.

## Data Availability Statement

The datasets generated for this study can be found in the PfTA atomic coordinates and structure factors were deposited in the Protein Data Bank (http://ww.pdb.org/) under accession ID 6S54.

## Author Contributions

FL-G and FP designed research. DR and RA performed research and analyzed the results. DR performed the bioinformatic research. DR, PS, and ID performed the crystallization and analyzed the data. DR, ID, FP, and FL-G wrote the paper.

### Conflict of Interest

The authors declare that the research was conducted in the absence of any commercial or financial relationships that could be construed as a potential conflict of interest.

## Abbreviations

PLP: pyridoxal-5-phosphate
TA: transaminase
PMP: pyridoxamine-5-phosphate
ωTA: ω-transaminase
TTN: total turnover number
HeTA: *Halomonas elongata* ω-transaminase
CvTA: *Chromobacterium violaceum* ω-transaminase
PfTA: *Pseudomonas fluorescens* ω-transaminase
RIN: residue interaction network
SHMT: serine hydroxymethyl transferase
DAAT: D-aminoacid transferase
*K*_*d*_: dissociation constant
T_1/2_: midpoint denaturation temperature.
