# Supplementary material for: Enhancing PLP-Binding Capacity of Class-III ω-Transaminase by Single Residue Substitution
Source: Front Bioeng Biotechnol. 2019 Oct 18;7:282. doi: 10.3389/fbioe.2019.00282 (PMC6813460; doi:10.3389/fbioe.2019.00282)
Supplement: Supplementary file 1 [file Data_Sheet_1.pdf]

Supplementary Material

1 Supplementary Data

The gene encoding an ω-transaminase from *Pseudomonas fluorescence* previously reported by Mutti *et al*. (2012)[1] was sequence optimized and ordered fused to a 6xHis tag (blue) and thrombin digestion (red underlined) site at its N-terminus. The synthesized sequence is;

MGSSHHHHHHSSGLVPRGSHMEFKRSNSNNKAWLKEHNTVHMMHPMQDPKALHEQRPLII
QSGKGVHITDVDGRRFIDCQGGLWCVNAGYGRREIIDAVTRQMEELAYYSLFPGSTNAPAIA
LSQKLTEVAAEEGMVKASFGLGGSDAVETALKIARQYWKLEGQPDKVKFVSLYNGYHGLN
FGGMSACGGNAWKSSYEPLMPGFFQVESPHLYRNPFTNDPEELAEICAQILERQIEMQAPGT
VAALIAEPIQGAGGVIVPPASYWPRLRQICDKYDILLIADEVITGLGRSGSLFGSRGWGVKPDI
MCLAKGISSGYVPLSATLVNSRVARAWERDAGFTSVYMHGYTYSGHPVSCAAALAAIDIVL
QENLAENARVVGDYFLEKLLILKDKHRAIGDVRGKGLMLAVELVKERATKEPFGPADAYPL
AISEACVNNGVMIRTIVNKLIISPPLTFTTEHVDEVIEVLDRAFVANPW

2 Supplementary Figures and Tables

Supplementary Table 1: Crystallographic data collection and refinement statistics.

	PfTA
**Data collection**	
Space group	$P2_12_12_1$
Cell dimensions	
a, *b*, *c* (Å)	92.02, 94.54, 242.22
Resolution (Å)	2.21
R_{pim}	0.131 (0.723)
$I / \sigma I$	3.9 (1.71)
CC1/2	0.973(0.589)
Completeness (%)	99.8 (88.07)
Redundancy	6.5 (6.5)
Wilson B-factor (Å^2)	28.7
No. of unique reflections	106548 (7759)
**Refinement**	
Resolution range (Å)	88.07-2.21
R_{work} / R_{free}	0.211/0.243
No. atoms	
Protein	13838
PLP	60
Water	328
B-factors (Å^2)	
Protein	65.35
PLP	56.13
Water	38.86
R.m.s. deviations	
Bond lengths (Å)	0.009
Bond angles (°)	1.062

*Values in parentheses are for highest-resolution shell.

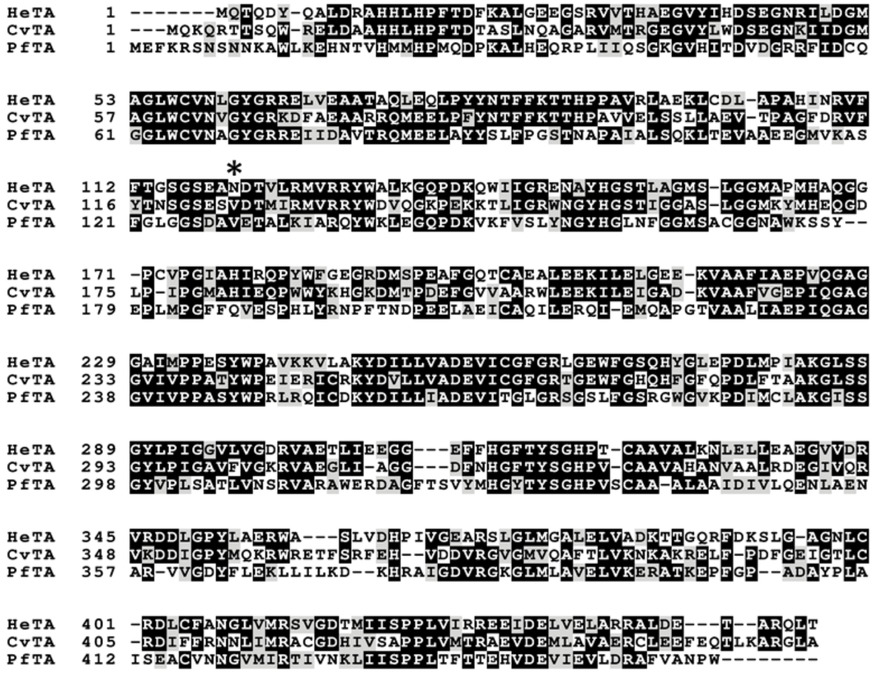

Supplementary Figure 1: **Multiple sequence alignment.** HeTA (CBV41788.1), CvTA (AAQ59697.1) and PfTA (PRA42448.1). The studied residue that plays a role in PLP binding in HeTA but not significantly in CvTA and PfTA is marked with a star above the sequence.

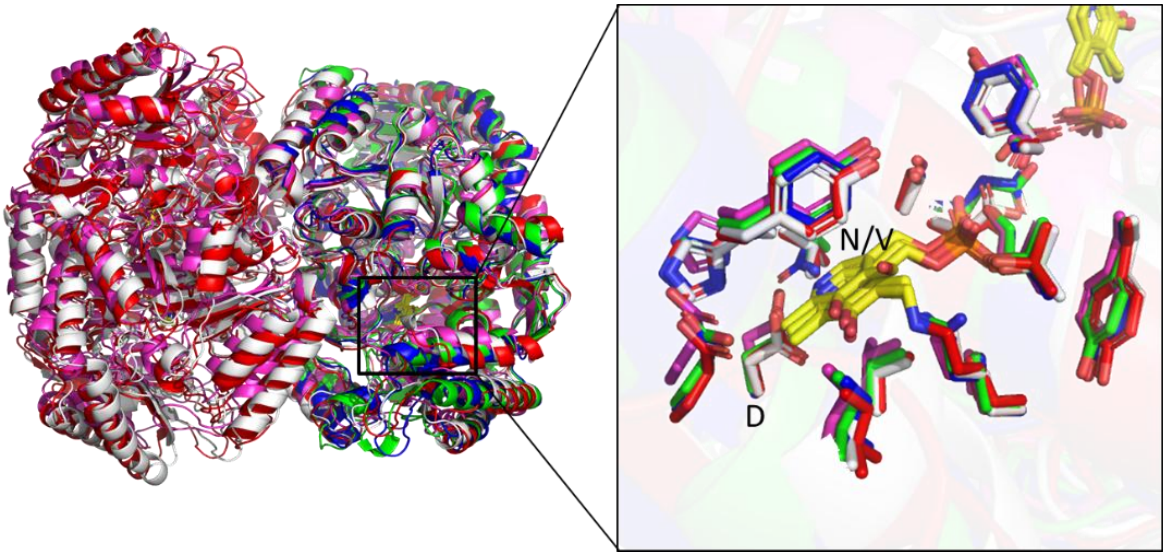

Supplementary Figure 2: Structural comparisons of HeTA, CvTA, PfTA, PaTA and PsTA. Superposition of dimeric HeTA (PDB: 6GWI; blue), dimeric CvTA (PDB:4A6T; green), tetrameric PfTA (PDB ID: 6S54; red), tetrameric PaTA (PDB ID:4B98; magenta) and tetrameric PsTA (PDB: 5LH9; white) structures (left) and close-up view showing the detail of their PLP binding cup (right). The PLP cofactor is depicted in yellow and the Asp residue and Asn (HeTA) or Val (CvTA, PfTA, PaTA) residues are labelled.

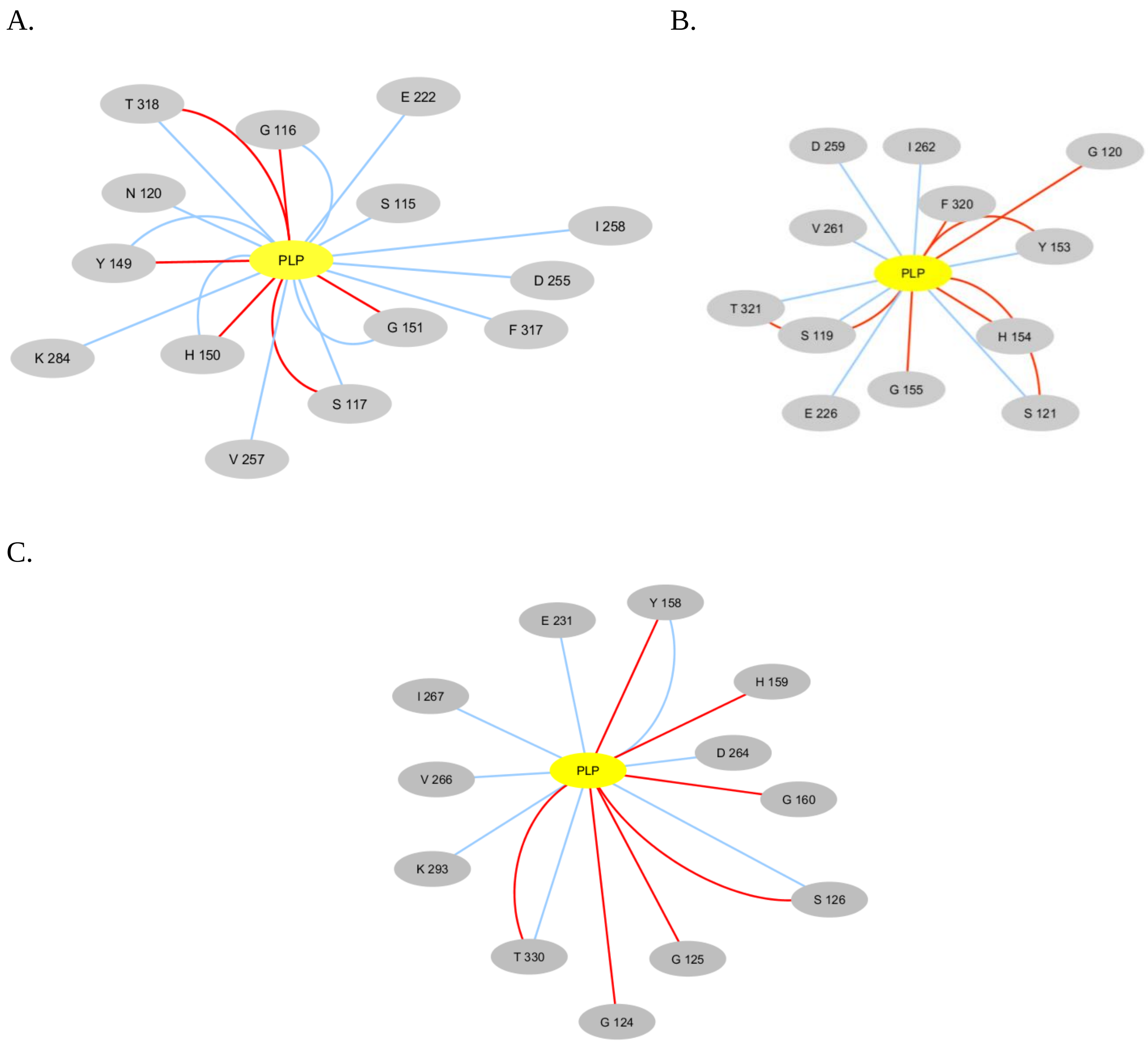

Supplementary Figure 3. PLP interactions in HeTA, CvTA and PfTA. The residue interaction network (RIN) for PLP in the HeTA (A), CvTA (B) and PfTA (C) structures is shown in diagram form. PLP is shown in yellow and, Main chain interactions are indicated in red and side chain interactions in light blue.

A. **B.**

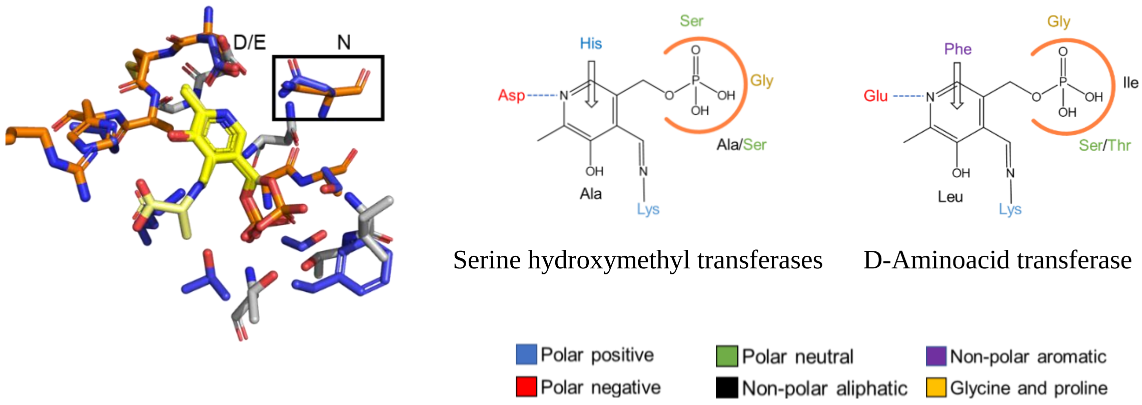

Supplementary Figure 4. Interactions of the PLP pyridine ring and phosphate moeities. **A.** Detail of the interaction of the pyridinium nitrogen with the aspartic or glutamic acid residue in HeTA (blue), DAAT (gray) and SHMT (orange). PLP is depicted in yellow and the serine bound for SHMT in light yellow. The acidic amino acid known to interact with PLP is labeled and the Asn marked with a black box. **B.** 2D representation of the key interactions in the PLP binding pocket. Hydrogen bonds are shown as a dashed blue line, π-π stacking interactions as an empty arrow and the phosphate binding cup is marked with an orange line. Amino acids are coloured as shown in the legend.

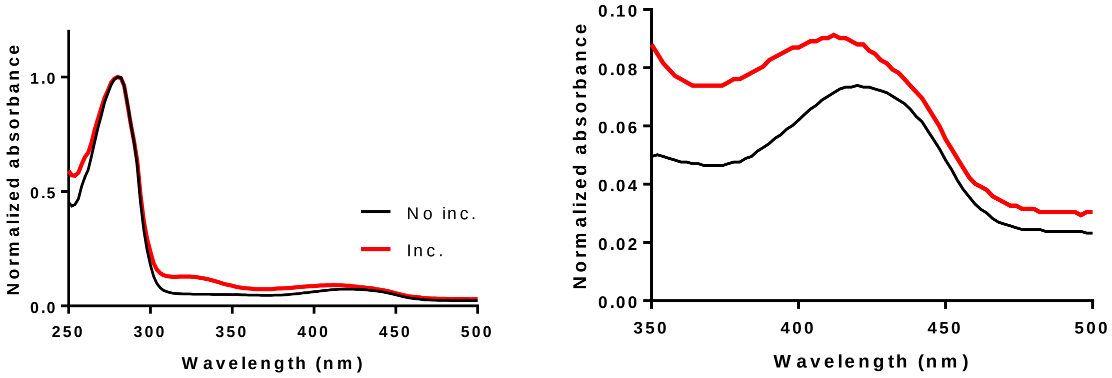

Supplementary Figure 5. Comparison of HeTA spectra before and after incubation with PLP. Spectra of pure HeTA enzyme before (black line) and after PLP incubation and elimination of free PLP with a PD-10 column (red line). **A.** Comparison of absorbance spectra in the range from 250 to 500 nm. **B**. Detail of the increase of the peak at 415 nm upon PLP incubation. Absorbances were normalized to the absorbance at 280 nm.

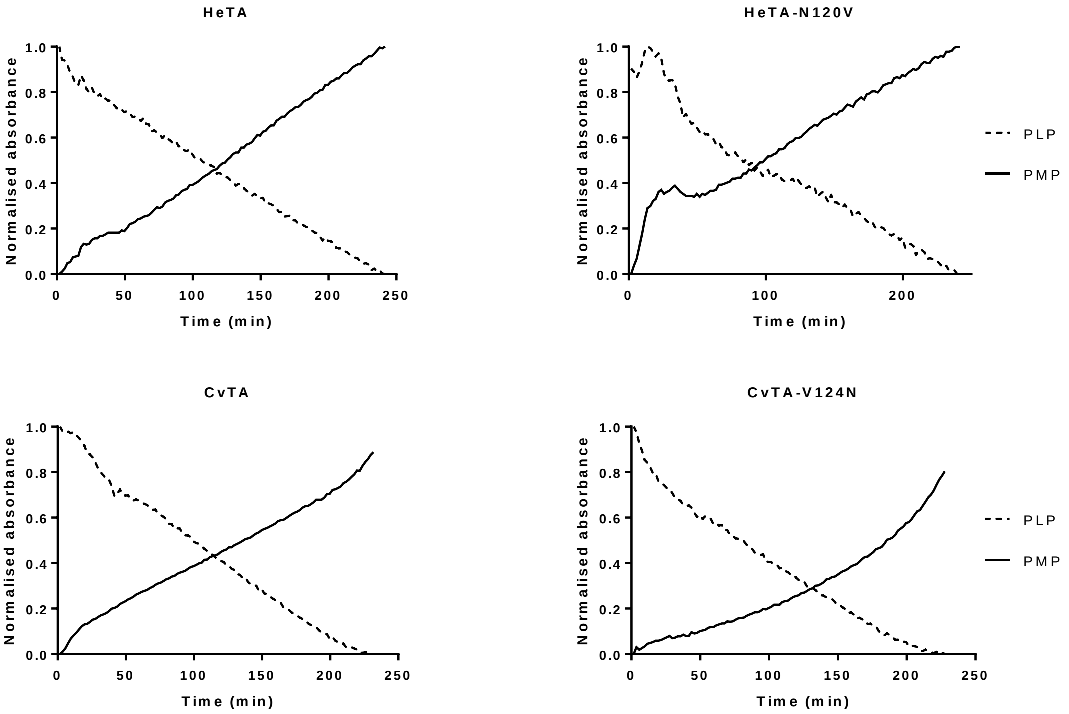

Supplementary Figure 6. PMP formation over time. From a reaction containing 0.5 mM enzyme, 50 mM benzylamine and 0.5 mM PLP, the formation of PMP (black line) was measured by following the increase of absorbance at 325 nm while PLP (dotted line) was monitored at 390 nm. Values of absorbance were normalized.

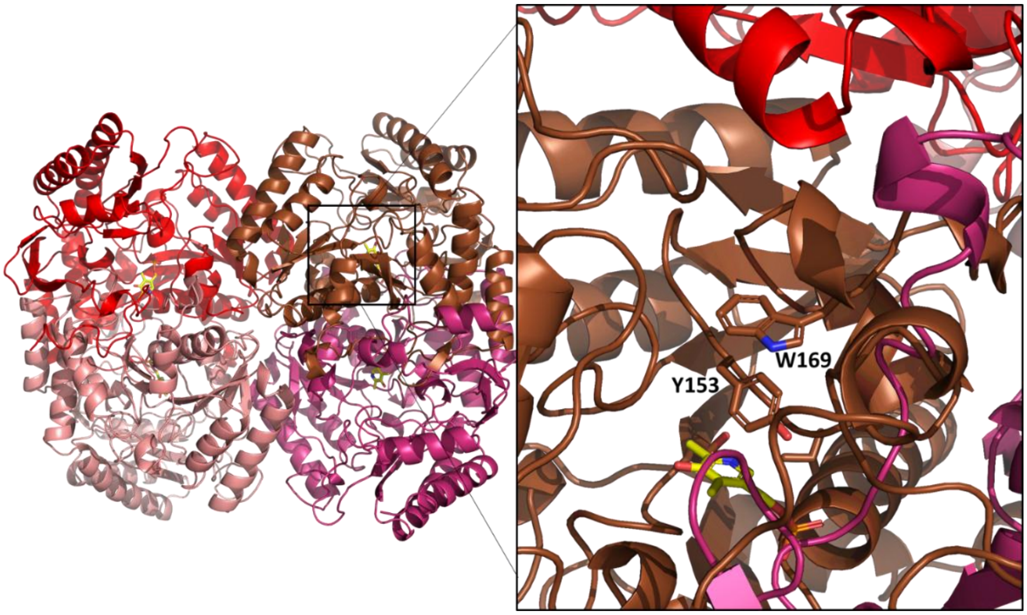

Supplementary Figure 7. Detailed view of the proposed loop anchor mechanism for PfTA. PfTA chains are shown in different red tone colours. Y178 and W194 are labelled that are hypothesised to play an important role in this mechanism.

References:

1. Mutti, F. G., Fuchs, C. S., Pressnitz, D., Turrini, N. G., Sattler, J. H., Lerchner, A., Skerra, A., and Kroutil, W. (2012) Amination of ketones by employing two new (S)-selective ω-transaminases and the his-tagged ω-TA from Vibrio fluvialis. *European J. Org. Chem.* 10.1002/ejoc.201101476
